# Effects of dapagliflozin on main pulmonary artery diameter in patients undergoing mitral valve replacement: a retrospective observational study

**DOI:** 10.3389/fcvm.2026.1759200

**Published:** 2026-02-19

**Authors:** Dong Wang, Pengxin Liu, Xiaodong Chen, Haoyu Hu, Haoze Wang, Zikang Zhang, Xu Liu

**Affiliations:** Department of Cardiac Surgery, The Affiliated Hospital of Qingdao University, Qingdao, China

**Keywords:** dapagliflozin, main pulmonary artery diameter, mitral valve replacement, pulmonary hypertension, SGLT2 inhibitor, vascular remodeling

## Abstract

**Objective:**

To investigate the effect of preoperative use of the sodium-glucose cotransporter 2 (SGLT2) inhibitor dapagliflozin on the perioperative main pulmonary artery (MPA) diameter in patients undergoing mitral valve replacement (MVR).

**Methods:**

This retrospective study analyzed 196 patients who underwent MVR at the Affiliated Hospital of Qingdao University between April 2020 and April 2025. Based on preoperative dapagliflozin use, patients were divided into a Dapagliflozin group (*n* = 39) and a Control group (*n* = 157). The baseline characteristics, perioperative echocardiographic parameters, and percentage changes in serological markers were compared between the two groups.

**Results:**

The baseline characteristics were balanced between the two groups. Postoperatively, the percentage change in MPA diameter was significantly lower in the Dapagliflozin group (−4.09% ± 16.77%) compared to the Control group (−2.21% ± 10.92%), with a statistically significant difference (*p* = 0.035). However, there were no significant differences in the percentage changes in pulmonary artery systolic pressure (PASP), left ventricular ejection fraction (LVEF), right ventricular function (TAPSE), right ventricular basal diameter (RVBD), or N-terminal pro-B-type natriuretic peptide (NT-proBNP).

**Conclusion:**

In patients undergoing mitral valve replacement, perioperative application of dapagliflozin was independently associated with a more stable and smaller main pulmonary artery diameter. This structural benefit was dissociated from changes in estimated pulmonary artery systolic pressure, suggesting that dapagliflozin may exert positive effects on the pulmonary artery via non-pressure-dependent direct vascular protective mechanisms.

## 1 Introduction

Pulmonary hypertension (PH) is one of the most common complications of left heart disease (1), particularly in patients with functional mitral regurgitation (MR) (1, 2), where it can lead to a significant increase in mortality (3). The 5th World Symposium on Pulmonary Hypertension defined PH as a resting mean pulmonary artery pressure (mPAP) ≥ 25 mmHg and proposed a classification system to guide clinical management by categorizing patients into five groups (1, 4–6). Among various PH groups, PH associated with left heart failure (HF) is by far the most common form, accounting for 65%–80% of PH cases (3). While targeted therapies for pulmonary arterial hypertension (PAH) are available, these treatments have not been fully evaluated or are not indicated, and may even be harmful in patients with PH related to left heart disease (LHD) (1–3).

The pathobiology of PH in left heart failure patients is complex, highly heterogeneous, and not fully understood (3). PH primarily results from the passive backward transmission of elevated left-sided filling pressures due to systolic or diastolic left ventricular dysfunction (7, 8). Furthermore, functional mitral regurgitation leads to increased left atrial pressure (LAP) and pulmonary artery pressure (PAP), often worsening during exercise (6, 9, 10). Current management focuses on optimizing heart failure medication and valve intervention (11), but there is a lack of specific targeted drugs impacting postoperative pulmonary artery pressure. This study specifically focused on patients with left ventricular ejection fraction (LVEF) < 50% to target the cohort in which systolic left ventricular dysfunction is the primary driver of secondary (functional) mitral regurgitation and the consequent post-capillary pulmonary hypertension (12). This selection criterion ensures pathophysiological homogeneity of the study population, allowing for a clearer investigation of drug effects within the context of established heart failure, and reduces confounding from including patients with markedly different etiologies (e.g., primary valvular disease with preserved systolic function).

Dapagliflozin is a highly potent, reversible, and selective SGLT2 inhibitor, globally indicated for the treatment of type 2 diabetes (T2D) (13). SGLT2 inhibitors reduce renal glucose reabsorption by inhibiting SGLT2 in the proximal tubule, promoting urinary glucose excretion and lowering blood glucose levels independently of insulin action (13–15). Given its antihyperglycemic, cardioprotective, and possible renoprotective properties with an overall favorable tolerability profile, dapagliflozin represents an important option for a broad patient population, irrespective of CVD history (15, 16). Emerging research suggests that dapagliflozin attenuates right ventricular structural remodeling and improves right ventricular function (14). This study was designed to investigate whether it could be beneficial for PH related to mitral valve disease.

Both mitral stenosis and regurgitation frequently lead to postcapillary pulmonary hypertension (7). According to the 2021 ESC/EACTS Guidelines for the management of valvular heart disease, reducing mitral regurgitation plays a crucial role in improving hemodynamics in patients with reduced ejection fraction (17). However, data indicate that even moderate elevation of pulmonary artery pressure can negatively impact postoperative outcomes after treatment (3).

## 2 Methods

### 2.1 Study design and patient enrollment

This single-center retrospective observational study included adult patients who underwent Mitral Valve Replacement (MVR) at the Affiliated Hospital of Qingdao University between April 2020 and April 2025. The study was approved by the hospital's Ethics Committee (Approval Number: QYFYWZLL30776), and the requirement for informed consent was waived due to the retrospective nature of the study. Patients in the Dapagliflozin group received standardized preoperative treatment according to our institutional protocol for managing concomitant heart failure, which consisted of oral dapagliflozin at a dose of 10 mg once daily. The preoperative treatment duration was at least 2 weeks, with a median duration of 4 weeks.

### 2.2 Inclusion and exclusion criteria

Inclusion Criteria: (a) Patients who underwent MVR at the Affiliated Hospital of Qingdao University; (b) Patients who underwent preoperative and postoperative echocardiographic and cardiac troponin assessment; (c) Left ventricular ejection fraction (LVEF) < 50%at baseline (to select a cohort with systolic left ventricular dysfunction, the primary driver of secondary mitral regurgitation and post-capillary pulmonary hypertension in this context) (d) Age ≥ 18 years. (e) Surgery was indicated for mitral regurgitation (MR).

Exclusion Criteria: (a) History of pulmonary hypertension (not caused by left heart dysfunction); (b) Emergency cardiac surgery; (c) Preoperative dapagliflozin use not meeting the standard regimen (i.e., a dose of 10 mg once daily for a minimum of 2 weeks prior to surgery); (d) Redo cardiac surgery; (e) Concomitant coronary artery bypass grafting (CABG); (f) Surgery involving artificial vessel replacement; (g) Significant hepatic or renal dysfunction. (h) Isolated predominant mitral stenosis.

### 2.3 Data collection

Baseline demographic and clinical data were recorded: age, sex, body mass index (BMI), blood pressure, history of hypertension, history of diabetes mellitus, operative time, beta-blocker use, angiotensin receptor-neprilysin inhibitor (ARNI) use, NT-proBNP level, left ventricular ejection fraction (LVEF), main pulmonary artery diameter (MPA), pulmonary artery systolic pressure (PASP), tricuspid annular plane systolic excursion (TAPSE), right ventricular basal diameter (RVBD), and NYHA functional class. Preoperative echocardiographic measurements were obtained during the routine pre-surgical evaluation within one week prior to mitral valve replacement (MVR). Postoperative measurements were conducted at a scheduled follow-up visit one week after hospital discharge, aiming to assess pulmonary vascular status in the early postoperative phase when patients were clinically stable.

### 2.4 Measurements

The diagnosis of pulmonary hypertension was confirmed by an experienced cardiologist. Transthoracic echocardiography was performed for each patient before and after surgery to assess ejection fraction, main pulmonary artery (MPA) diameter, pulmonary artery systolic pressure (PASP), tricuspid annular plane systolic excursion (TAPSE), and right ventricular basal diameter (RVBD). The MPA diameter was specifically measured in the parasternal pulmonary artery long-axis view at end-systole, just proximal to the bifurcation. All measurements were performed following a standardized institutional protocol aligned with the recommendations of the American Society of Echocardiography (ASE) to ensure consistency and reproducibility. To minimize inter-operator variability and subjective bias, measurements of MPA diameter were performed independently by two experienced cardiac sonographers who were blinded to patient group allocation. The inter-observer agreement was assessed by calculating the intraclass correlation coefficient (ICC) using a two-way random-effects model for absolute agreement. The resulting ICC was (0.93), indicating excellent reliability. For statistical analysis, the average of the two measurements was used.

### 2.5 Outcome measures

The primary clinical outcome was the percentage change in echocardiographic parameters from preoperative to postoperative measurements. Secondary clinical outcomes included changes in biomarker levels and other echocardiographic variables.

### 2.6 Statistical analysis

All statistical analyses were performed using SPSS version 26.0. Continuous variables conforming to a normal distribution are presented as mean ± standard deviation and were analyzed using the t-test; non-normally distributed variables are presented as median and were analyzed using the Wilcoxon signed-rank test. Categorical data are presented as numbers and were compared using the Chi-square test or Fisher's exact test. Pre- and postoperative results were compared using paired *t*-tests. A *p*-value < 0.05 was considered statistically significant for all analyses.

## 3 Results

**Figure 1:**
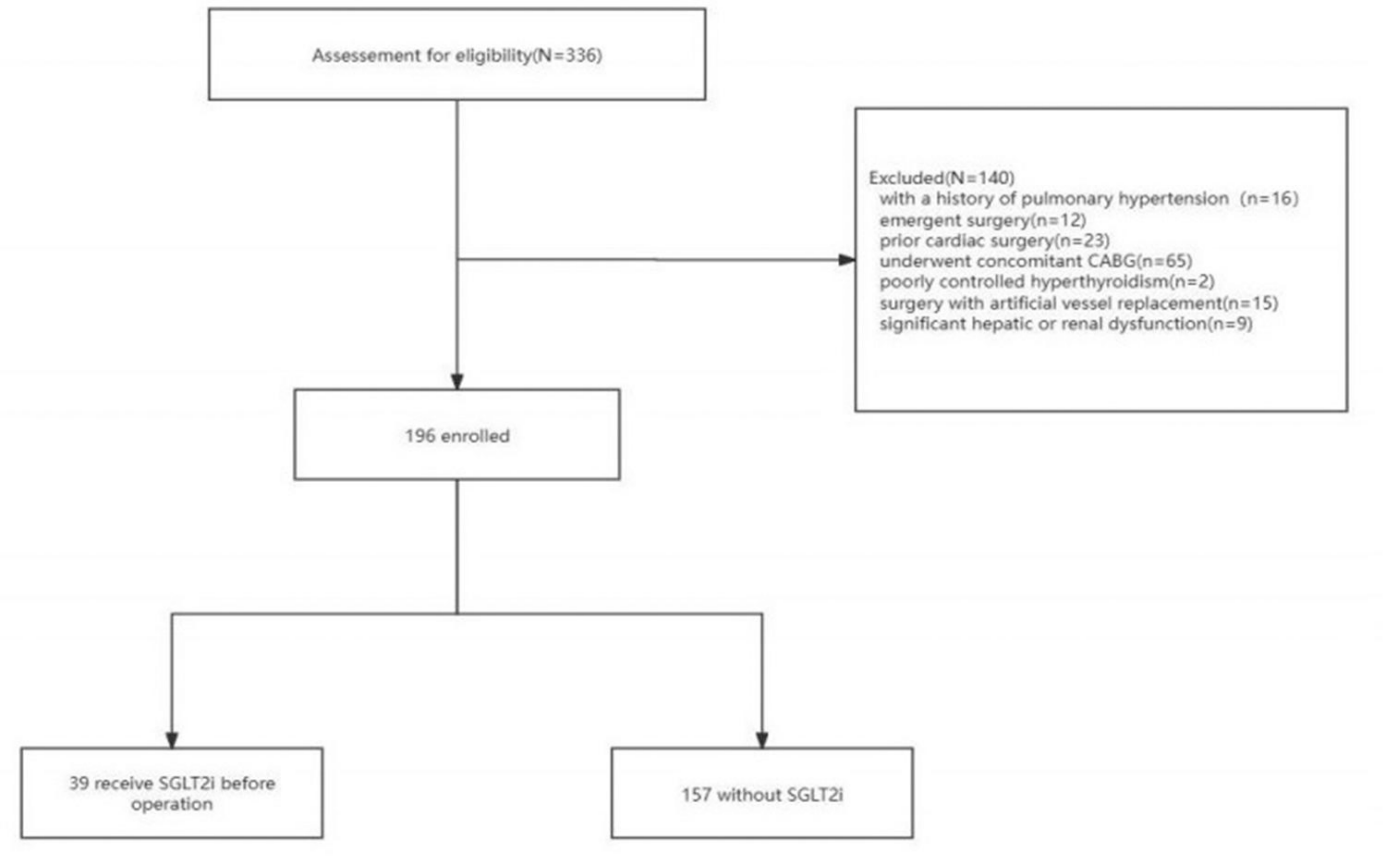
Flowchart of patient selection and enrollment.

A total of 336 medical records of patients who underwent MVR were assessed. After applying exclusion criteria (16 with a history of PH, 12 emergent surgeries, 23 prior cardiac surgeries, 65 concomitant CABG, 2 poorly controlled hyperthyroidism, 15 artificial vessel replacements, 9 with significant hepatic/renal dysfunction), 196 patients met the inclusion criteria. Among them, 39 patients received standardized dapagliflozin treatment as part of the preoperative plan. The patient flow diagram is shown in Figure 1.

Among the 196 enrolled patients, no significant differences were observed between the Dapagliflozin and Control groups regarding age, sex distribution, BMI, pulse pressure, preoperative NT-proBNP levels, NYHA class, history of hypertension, or history of diabetes mellitus.

No statistically significant differences were found in preoperative medication use, including beta-blockers and ARNI, between the Dapagliflozin and Control groups. Similarly, preoperative echocardiographic parameters, including LVEF, MPA diameter, PASP, TAPSE, and RVBD, showed no statistically significant differences between the groups. These data indicate that the baseline clinical status and cardiac function of the two groups were essentially comparable, providing a reliable basis for comparing the effects of the intervention. Detailed data are shown in Table 1.

**Table 1:** Baseline demographic, clinical, and echocardiographic characteristics of the study population stratified by dapagliflozin use.

Items	Dapagliflozin	Controls	*P*-value
Age(years)	61.4 ± 9.2	60.7 ± 9.7	0.727
Male/female	30/9	97/60	0.076
BMI(kg/m^2^)	25.8 ± 3.5	25.0 ± 3.7	0.268
Pulse Pressure(mmHg)	46.1 ± 12.2	49.6 ± 15.0	0.356
Hypertension	12	69	0.135
Diabetes mellitus	7	31	0.800
Operative time (min)	244 ± 84	256 ± 85	0.142
Beta-blockers	21	91	0.642
ARNI	7	14	0.103
Preop. NT-proBNP(pg/mL)	2,078.0 ± 3,108.8	1,862.6 ± 2,451.4	0.118
LVEF(%)	45.8 ± 4.8	46.6 ± 4.4	0.269
MPA(mm)	25.9 ± 3.5	25.2 ± 3.5	0.201
PASP(mmHg)	51.4 ± 22.2	46.8 ± 15.9	0.365
TAPSE(mm)	19.1 ± 3.7	20.2 ± 2.9	0.070
RV Basal Diameter(mm)	57.9 ± 8.8	56.1 ± 8.0	0.558
NYHA III-IV			
III	15	56	0.745
IV	24	101	

Analysis of the percentage change in postoperative parameters (Table 2) revealed differences between groups: The percentage change in MPA diameter from preoperative to postoperative was −4.09 ± 16.77 in the Dapagliflozin group compared to −2.21 ± 10.92 in the Control group, showing a statistically significant difference (*p* = 0.035). The percentage change in MPA diameter was significantly reduced in the Dapagliflozin group compared to the Control group. Dapagliflozin treatment did not cause significant changes in other cardiac aspects. Left ventricular ejection fraction remained stable pre- and postoperatively in both groups without significant changes. TAPSE was also similar between the two groups, and changes in PASP were not significant.

**Table 2:** Perioperative percentage change in cardiac structure and function parameters: dapagliflozin vs. Control.

Items	Dapagliflozin	Controls	*p*
Percentage change in MAP (%)	−4.09 ± 16.77	−2.21 ± 10.92	0.035
Percentage change in LVEF (%)	0.02 ± 0.13	−0.01 ± 0.11	0.174
Percentage change in TAPSE (%)	−0.01 ± 0.30	0.02 ± 0.32	0.381
Percentage change in PASP (%)	−0.27 ± 0.37	−0.29 ± 0.23	0.812
Percentage change in RVBD (%)	0.01 ± 0.14	0.05 ± 0.19	0.264
Percentage change in NT-proBNP (%)	1.13 ± 4.65	4.99 ± 62.35	0.104

**Figure 2:**
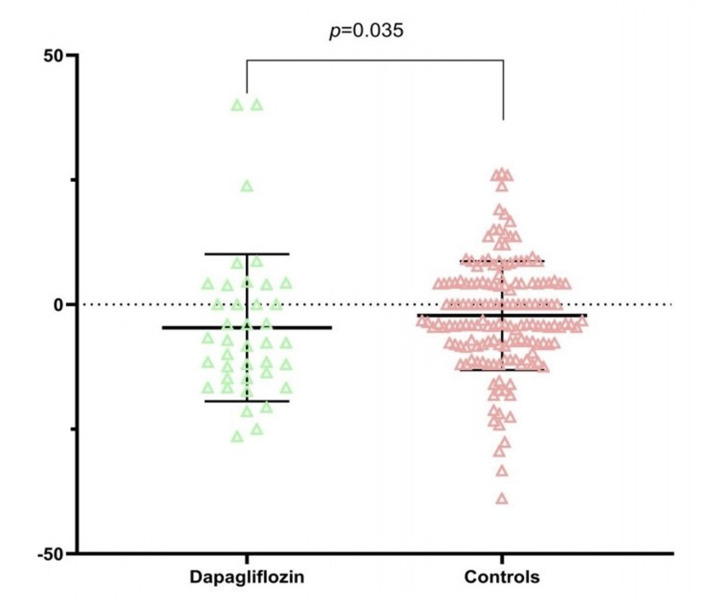
Comparison of main pulmonary artery (MPA) diameter between the dapagliflozin and control groups.

The main finding of this study pertains to the structure of the main pulmonary artery (MPA). As shown in the box plot (Figure 2), the postoperative MPA diameter was significantly smaller in the Dapagliflozin group compared to the Control group. Intergroup comparison confirmed that this difference was statistically significant (**p = 0.035**). It is noteworthy that despite the significant difference in MPA diameter, the percentage change in estimated PASP showed no statistical difference between the two groups (*p* = 0.812).

## 4 Discussion

The main finding of this study reveals a thought-provoking phenomenon: preoperative use of the SGLT2 inhibitor dapagliflozin in patients undergoing cardiac surgery was significantly associated with a smaller percentage change in main pulmonary artery (MPA) diameter postoperatively. Specifically, the MPA diameter in the Dapagliflozin group remained more stable (percentage change: −4.09%), whereas the Control group showed a relative trend towards greater dilation (percentage change: −2.21%), a difference that was statistically significant (*p* = 0.035). Crucially, this structural benefit was independent of changes in other hemodynamic and functional parameters, providing a new perspective for understanding the protective effects of SGLT2 inhibitors on the cardiovascular system.

### 4.1 Core finding: SGLT2 inhibitor and stabilization of pulmonary artery structure

Our data consistently indicate that dapagliflozin treatment is associated with a smaller MPA diameter (Figure 2) and significantly inhibits the perioperative percentage change in MPA diameter. After excluding significant baseline imbalances (Table 1) and interference from other cardiac functional parameters (such as LVEF, TAPSE, RVBD), this specific effect on pulmonary artery structure is particularly prominent. It is noteworthy that this effect was observed in a cohort specifically selected for having LVEF < 50%, representing patients with systolic dysfunction as the core mechanism for secondary mitral regurgitation and elevated left-sided filling pressures. MPA diameter is an important morphological indicator reflecting long-term pulmonary artery load and the intrinsic properties of the vascular wall (7). Its dilation is typically recognized as a surrogate imaging marker for pulmonary hypertension and vascular remodeling (7). Therefore, our findings are consistent with the possibility that dapagliflozin may contribute to stabilizing pulmonary artery structure and inhibiting adverse remodeling during the perioperative stress state.

### 4.2 Mechanism exploration: “pressure-structure decoupling” and the pleiotropic effects of SGLT2 inhibitors

We acknowledge the reviewer's important point that classical pulmonary vascular remodeling is a chronic process. The MPA diameter change observed in our study, following a median preoperative treatment period of 4 weeks, is more likely to reflect rapid modulation of hemodynamics and vascular function by dapagliflozin rather than chronic structural remodeling. Recent evidence from major heart failure trials confirms that dapagliflozin induces rapid hemodynamic and clinical benefits across the spectrum of ejection fraction (18). Potential mechanisms underlying this early effect may include: (1) rapid preload reduction through natriuresis and diuresis, thereby decreasing passive distending forces on the pulmonary arterial wall; and (2) pleiotropic vascular actions, such as anti-inflammatory and endothelial-improving effects, which may optimize vascular compliance in the short term. Thus, the “structural” stabilization we report may represent an early morphological consequence of these functional and hemodynamic improvements.

The most insightful finding of this study is the dissociation between the improvement in MPA and pulmonary artery pressure, i.e., “pressure-structure decoupling”. Although the MPA diameter was smaller and more stable in the Dapagliflozin group, the estimated pulmonary artery systolic pressure (PASP) showed no significant difference between the groups (percentage change *p* = 0.812). This raises the hypothesis that the benefit of dapagliflozin may not be primarily achieved through direct reduction of pulmonary artery pressure, but rather by pressure-independent mechanisms that could confer vascular protection.

We propose the following mechanistic hypotheses to explain this phenomenon:

Direct Anti-inflammatory and Anti-fibrotic Vascular Protection: Growing evidence indicates that SGLT2 inhibitors possess significant anti-inflammatory and anti-fibrotic properties (14, 19). Heart failure and surgical stress can trigger systemic and pulmonary vascular inflammatory responses, driving vascular matrix degradation and fibrosis, leading to vascular dilation and reduced compliance (20, 21). Dapagliflozin may directly inhibit these pathological processes (8), enhancing the elasticity and structural integrity of the pulmonary arterial wall, thereby resisting dilative remodeling.
Indirect Effect via Improved Left Heart Filling Pressures: The hypothesis of an indirect benefit mediated by reduced left heart filling pressures is proposed primarily based on extrapolation from large randomized controlled trials where SGLT2 inhibitors, including dapagliflozin, have demonstrated rapid reduction in pulmonary capillary wedge pressure (PCWP).Although PASP remained unchanged, SGLT2 inhibitors have been proven to effectively reduce left ventricular filling pressures through their diuretic and natriuretic effects (19). Reduced left atrial pressure (LAP) can retroactively alleviate congestion in the pulmonary venous system and the passive tension in the pulmonary arteries (3). This reduction in “upstream” pressure may provide a “decompressed” environment for the MPA, inhibiting its dilation, while the PASP estimated via tricuspid regurgitation may be insensitive to this change.
Improved Endothelial Function and Vascular Tone Regulation: SGLT2 inhibitors have been shown to improve endothelial function, restore nitric oxide (NO) bioavailability, and reduce oxidative stress (19). Improved endothelial function can optimize vascular tone and adaptability, potentially improving vascular stiffness and maintaining a healthier diameter without significantly altering mean pressure.

### 4.3 Clinical implications and future directions

This study is the first to reveal an association between SGLT2 inhibitors and improved pulmonary artery structure in a perioperative cardiac surgery population, adding a new dimension to their organ protection profile—pulmonary artery protection. The finding of a smaller postoperative MPA diameter is clinically significant. As the main conduit of the right ventricular (RV) outflow tract, the MPA is a primary determinant of RV afterload. A smaller diameter implies reduced wall tension and, consequently, lower RV afterload. Given the particular sensitivity of the thin-walled RV to afterload changes, attenuating this load during the postoperative period may facilitate RV functional recovery and adaptation.

In cardiac surgery, right ventricular dysfunction is an important prognostic factor. The structure and function of the pulmonary artery are important contributors to right ventricular afterload, and the main pulmonary artery (MPA) diameter is a recognized surrogate imaging marker for these underlying changes (6, 10, 22). This is supported by growing imaging evidence linking MPA diameter to outcomes. For instance, recent studies have identified MPA diameter as an independent prognostic imaging marker for RV dysfunction (23). Although derived from different clinical contexts (e.g., acute pulmonary embolism), the underlying physiological principle is consistent: pulmonary artery dilation reflects chronic pressure or volume overload. Therefore, inhibiting early postoperative MPA dilation, as observed with dapagliflozin, may indicate a more favorable RV-pulmonary artery coupling state. Inhibiting pathological pulmonary artery dilation may provide a more favorable “downstream” environment for the right ventricle, which could have profound implications for patients' long-term outcomes.

### 4.4 Study limitations

It must be noted that this is an observational study and cannot establish causality. Furthermore, pulmonary artery pressure was indirectly estimated by echocardiography; future studies employing direct measurement via right heart catheterization, particularly measuring mean pulmonary artery pressure and pulmonary capillary wedge pressure, could more precisely reveal the hemodynamic mechanisms. Additionally, although the preoperative and postoperative assessment time windows were standardized (within one week before surgery and one week after discharge), the exact timing of echocardiographic measurements was not uniform across all patients, which is a common limitation in retrospective studies. Future prospective studies with strictly fixed time points would be valuable. Finally, we acknowledge that the sample size of this study is limited and the conclusions require validation by larger prospective studies. Furthermore, while we propose plausible mechanisms, they remain hypothesis-generating due to the lack of direct measures of pulmonary vascular properties or left-sided filling pressures. The observed change in MPA diameter is thus best interpreted as a promising surrogate endpoint, with its precise mechanistic underpinnings awaiting future confirmation.

## 5 Conclusion

This study demonstrates that perioperative application of the SGLT2 inhibitor dapagliflozin in patients undergoing cardiac surgery is independently associated with a more stable and smaller main pulmonary artery (MPA) diameter, and this structural benefit is dissociated from changes in estimated pulmonary artery systolic pressure (PASP). This strongly suggests that the protective effect of dapagliflozin on the pulmonary artery may stem from direct anti-vascular remodeling and indirect optimization of the pulmonary artery environment through improved left heart loading—“non-pressure-dependent” mechanisms. This discovery deepens our understanding of the pleiotropic effects of SGLT2 inhibitors and provides an innovative theoretical basis for their potential application in the field of pulmonary vascular disease.

## Data Availability

The datasets generated and analyzed during this study are not publicly available due to the following restrictions: (i) patient privacy and confidentiality — the data contain detailed, sensitive clinical information of individual patients. Public dissemination would violate the privacy rights of the participants and the confidentiality agreements under which the data were collected, (ii) institutional data policy — the data are the property of the Affiliated Hospital of Qingdao University and are governed by its specific data management and security policies, which prohibit the public sharing of raw clinical data, (iii) conditional access: — in accordance with principles of research transparency, de-identified data (with all personal identifiers removed) may be made available to qualified researchers for the purpose of reproducing or validating the study's findings. Such requests must be submitted in writing to the corresponding author and will require explicit approval from the Ethics Committee of the Affiliated Hospital of Qingdao University (Approval Number: QYFYQZLL30197). A formal data sharing agreement may be necessary. Requests to access these datasets should be directed to Xu Liu, peterxuxu@126.com.
